# Quality of life and associated factors for community health workers in the context of the COVID-19 pandemic in northeastern Brazil

**DOI:** 10.1038/s41598-024-63828-9

**Published:** 2024-06-10

**Authors:** Franklin Delano Soares Forte, Neiva Francenely Cunha Vieira, Sidney Feitoza Farias, Isabella Lima Barbosa Campelo, Marcia Castro, Aisha Khizar Yousafzai, Anya Pimentel Gomes Fernandes Vieira-Meyer

**Affiliations:** 1 Departamento de Clínica e Odontologia Social, Centro de Ciências da Saúde, Universidade Federal da Paraíba, Campus Universitário I, Castelo Branco I, João Pessoa, Paraíba Zip-code 58051-900 Brazil; 2 Faculdade de Farmácia, Odontologia e Enfermagem, Universidade Federal do Ceará, Rua Alexandre Baraúna 1115, Rodolfo Teófilo, Fortaleza, Ceará Zip-code 60430-160 Brazil; 3 Departamento de Saúde Coletiva, Instituto Aggeu Magalhães - Fiocruz Pernambuco, Av. Prof. Moraes Rego, s/n, Cidade Universitária, Recife, Pernambuco Zip-code 50670-420 Brazil; 4 Unifanor Wyden Rua Antônio Gomes Guimarães, 150 Papicu, Fortaleza, Ceará Zip-code: 60191-195 Brazil; 5 Department of Global Health and Population Harvard T.H. Chan School of Public Health, 677 Huntington Avenue, Boston, MA 02115 USA; 6 Fundação Oswaldo Cruz – Ceará, R. São José, s/n, Eusébio, Ceará Zip-code 61760-000 Brazil; 7 Centro Universitário Christus, R. João Adolfo Gurgel, 133 - Cocó, Fortaleza, Ceará zip code 60190-180 Brazil

**Keywords:** Community health workers, COVID-19, Quality of life, Violence, Primary care, Health policy, Health services, Quality of life

## Abstract

In this study, we evaluated the quality of life (QoL) and associated factors of community health workers (CHWs) in different urban settings as a contributor to the preparedness of Brazilian primary care for future sanitary emergencies. The sample included 1935 CHWs from 4 state capitals and 4 inland municipalities in northeastern Brazil. Information was collected on QoL (WHOQOL-BREF), work routines, sociodemographics, direct and indirect exposure to violence, general self-efficacy, social support (MSPSS), mental health (SRQ-20) and coronavirus anxiety. The data were subjected to multiple linear regression analysis (α = 5%). In the state capitals, the factors associated with loss of QoL were poor mental health, lack of training, uncertainty about occupational biosafety, and lack of adaptation of services to tend to patients diagnosed with COVID-19. Among the inland municipalities, the main factors were coronavirus anxiety, poor mental health, lack of adaptation of services, lack of training, and lack of personal protective equipment (PPE). A high MSPSS (family and friends) score and self-efficacy had positive impacts on QoL in both urban settings. Our results highlight the need for investment in permanent education, PPE, social support, and mental health care for CHWs.

## Introduction

The Brazilian Unified National Health System (SUS) represents a significant public health policy, structured on the primary health care (PHC) model and implemented, at the local level, by the Family Health Strategy (FHS), which provides multiprofessional care^1^. PHC played an important role in the Brazilian response to COVID-19, which required timely readjustments to the care provided by public health care teams (ESFs), including changes in protocols, patient flow and work routines^2^.

FHS is the primary strategy for consolidating and expanding PHC within Brazil. It includes the implementation of democratic and participatory health care and management practices through multidisciplinary teams covering defined populations and territories. In addition, the FHS considers each human being according to their singularity and socio-cultural context in a quest for comprehensive care^3^. The FHS multiprofessional team is comprised of a doctor, a nurse, a dentist, health auxiliaries and community health workers (CHWs), who are trained community members that reside and work within the territory served by the FHS, so they are familiar with the context of people's lives, which greatly strengthens the bonds between the community and the Primary Health Care (PHC) teams. CHWs primarily engage in providing guidance and support for their team’s health professionals and conducting health promotion activities and home visits to assist and collect information on the health and living standards of families. Their community presence facilitates residents’ access to health services and strengthens the bond between families and the FHS^4^.

The incidence of urban violence has increased markedly in various regions of the world, including Northeast Brazil. Among the 50 most violent cities in the world in 2023, there are ten Brazilian northeast municipalities, including six of the nine state capitals^5^, three of which were researched in the present article. The high level of community violence and low social indicators (e.g., high illiteracy, low urban development index and high percentage of the population living in poverty) in the Northeast denote the vulnerable daily reality of this region^6^. Furthermore, this vulnerability scenario was worsened by COVID-19. In the first year of the Covid-19 pandemic, this region, which accounts for 27% of the Brazilian population, recorded a third of the cases (34%) and deaths (32%) of COVID-19, demonstrating the great impact of the pandemic in the Brazilian northeast^7^.

It has been demonstrated that violence, the Covid-19 pandemic, and other stressors are associated with the process of CHW work and mental health^4,8–11^. Nevertheless, not much is known on the CHWs quality of life on this stressed context.

The work setting of CHWs has changed drastically as long-standing social, environmental and cultural dynamics have been reshaped by new circumstances associated with lockdown restrictions, a lack of information about the manifestations of the disease, increased mortality, and a loss of quality of life (QoL) for the population at large and for health professionals in particular^12,13^.

The changes in work routines brought about by sanitary emergencies affected CHWs’ personal and professional lives and quality of life due to a complex constellation of sociodemographic, economic, environmental, cultural and health factors, with all their subjectivities and intersubjectivities. Quality of life may be defined in many ways. According to the World Health Organization (WHO), 'Quality of Life' refers to an individual's perception of their position in life, considering their socioeconomic context, habits, customs, values, goals, expectations, and concerns^14^.

QoL can be assessed in different ways and with different tools, one of which is the WHOQOL-BREF^15^, developed and recommended by the World Health Organization (WHO). Frontline health professionals are particularly prone to a decrease in QoL due to anxiety, depression and burnout, aggravated by factors of social unrest and violence in the territory^4,16^.

The availability of social support for health workers in terms of instrumental and emotional factors and social interaction has been shown to preserve QoL in both the occupational setting and the family setting^17–19^, which has become increasingly evident during recent pandemics^20,21^. Studies published before the pandemic identified sex, age, schooling, workplace, family income, smoking, psychological aspects and satisfaction as determinants of QoL^22^.

Despite a number of similar actions and measures taken in the early days of the pandemic, the response to health emergencies varied among municipalities according to local socioeconomic, demographic, epidemiological, social and political factors and the capacity and resilience of the public health care infrastructure^7,23,24^.

It is important to investigate to what extent the CHWs’ QoL was affected during this period, taking into account the context of violence in the territories and the sociodemographic and economic situation of each municipality. A discussion on this issue can contribute new knowledge and help create interventions for the promotion of QoL among CHWs, identify vulnerable groups, design health care policies and plan actions. In addition, the research conducted during the pandemic and the questions raised are important aids in preparing CHWs for future health emergencies.

The purpose of this study was to assess the QoL of CHWs from Northeast Brazilian state capitals and inland municipalities, as well as factors related to mental health, socioeconomic variables and the primary care setting.

## Methods

This was a cross-sectional, multicenter study based on information collected in four Brazilian state capitals (Fortaleza/Ceará, João Pessoa/Paraíba, Recife/Pernambuco, Teresina/Piauí) and four inland municipalities in the state of Ceará (Crato, Juazeiro do Norte, Barbalha, Sobral) between April and August 2021. The sampling locations were chosen to obtain a representative picture of the factors associated with CHW QoL in both central and inland urban settings. The Northeast is a region with high social vulnerability and political, economic, territorial inequalities and has been one of the worst affected by the pandemic^7^. Primary health care coverage in the municipalities studied ranges from 58.12% (Recife) to 100% (Sobral)^25^; the population from 59 thousand (Barbalha) to 2.4 million (Fortaleza)^26^ and the HDI of 0.683 (Barbalha) to 0.772 (Recife)^27^.

In 2020, according to the Health Ministry’s e-management system, the eight sampled cities employed a total of 7909 CHWs^28^. Using a simple sample calculation based on the number of CHWs in each municipality, a sample error of 5%, a 95% confidence level, and a homogeneous distribution (80/20) of the sampled population, we arrived at a minimum sample size of 1879 CHWs. However, the actual number of CHWs interviewed for the study (n = 1935) exceeded this number.

To be eligible, CHWs had to be active members of ESF teams during the study period and have at least one year of experience as CHWs. Participation was spontaneous upon invitation. CHWs on leave or vacation were excluded.

To standardize the data collection procedure, the collectors were trained using a manual prepared for the purpose. The training started with the theoretical aspects of each stage. Due to the sanitary conditions under which the study was conducted, we employed the biosafety protocols established in the GVIMS/GGTES/ANVISA directive 04/2020 in addition to complying with the standard ethical requirements of research involving human subjects.

The self-reporting questionnaire (SRQ-20) collected information on sociodemographics, work, psycho-emotional symptoms, experience with COVID-19, general self-efficacy (GSE), multidimensional perceived social support (MSPSS), coronavirus anxiety, exposure to violence in territories, work issues in territories, and the WHOQOL-BREF (dependent variable).

The WHOQOL-BREF is widely used to assess QoL and contains 26 items covering 4 domains: physical health, psychological health, social relationships, and the environment. Each item is scored on a Likert scale, with higher scores indicating a better QoL^29,30^.

Focusing on psychoemotional symptoms, the SRQ-20 has been extensively used in Brazilian studies to measure indicators of common mental disorders (CMDs), especially in occupational settings, and is an important mental health screening tool that uses ≥ 7 as the cutoff^31^. Ranging from 0 to 20, the SRQ score corresponds to the number of affirmative answers to 20 dichotomous questions. The instrument has been validated for Brazil^32^.

The Coronavirus Anxiety Scale (COVID-19) is used to assess anxiety induced by concerns about COVID-19^33^, with higher scores indicating greater anxiety. The instrument features five questions scored on a scale from 0 to 4, ranging from 0 to 20.

The MSPSS was introduced by Zimet et al.^34^ and has been validated for Brazilian Portuguese^35^. It is based on the perception that people in one’s social sphere provide affective and material resources, a sense of belonging, and a supportive network^36,37^. Social support can alleviate distress during times of uncertainty, crisis, anxiety and tension^38^. The instrument features 12 items, covering three factors (family, friends and significant others which look at the presence of a special person in time of need as a source of comfort, to share joys and sorrows, and care about feelings)^39^, and is scored using a Likert scale^36^.

The collected data were analyzed using the statistical software R, with the level of significance set at 5% (*p* < 0.05). The outcome variable was QoL, expressed as the total WHOQOL-BREF score. In addition, multiple linear regression analysis with backward elimination was performed using Akaike’s information criterion due to the exploratory nature of the model. As with the correlation analyses, the data from the state capitals and the inland municipalities were analyzed separately.

All the participating CHWs provided informed written consent. Filed under #4.587.955, the study protocol was approved by the Internal Review Board of Ceará State University (UECE).

### Ethics approval and consent to participate

This research was approved by the Research Ethics Committee of Ceará State University (UECE) (authorization #4.587.955). Written informed consent was obtained from all the participants in the study. All procedures were performed in accordance with relevant guidelines.

## Results

The total sample consisted of 1935 CHWs, with a predominance of females (82.76%). The average age was 46 years (range: 23–72). Most had children (80.98%), but no spouse or partner (58.21%) was identified as Catholic (65.85%), and most were indigenous/brown (71.84%). Nearly half (47.26%) had completed high school and earned up to 2 minimum wages (at the time of writing, 1 minimum wage was equivalent to USD ~ 260). Table 1 shows the statistical analysis of the sociodemographic variables.

**Table 1:** Sociodemographic variables of community health workers (CHWs) according to location (state capitals vs. inland municipalities).

Variables	State capitals		Inland municipalities	
	Mean (SD) or n(%)	95% CI	Mean (SD) or n(%)	95% CI
Age	46.92 (8.13)	46–47	45.24 (9–39)	44–46
Sex				
Female	875 (80.42)	78–83	484 (87–36)	84–90
Male	213 (19.58)	17–22	70 (12–64)	10–16
City				
Barbalha			80 (14–44)	12–18
Crato			108 (19–49)	16–23
Fortaleza (capital)	298 (27.34)	25–30		
João Pessoa (capital)	259 (23.76)	21–26		
Juazeiro do Norte			180 (32–49)	29–37
Recife (capital)	291 (26.70)	24–29		
Sobral			186 (33–57)	30–38
Teresina (capital)	242 (22.0)	20–25		
Time of residence in neighborhood	31.03 (12.98)	30–32	30.92 (14–71)	30–32
Marital status				
Has no spouse/partner	613 (56.24)	53–59	344 (62–9)	58–66
Has spouse/partner	477 (43.76)	41–47	210 (37–91)	34–42
Children				
No	204 (18.78)	17–21	108 (19–49)	16–23
Yes	882 (81.2)	79–83	446 (80–51)	77–84
Religion				
Missing	146 (13.39)	11–16	34 (6.14)	4.3–8.6
Catholic	526 (48.26)	45–51	438 (79.06)	75–82
Spiritist	36 (3.30)	2.4–4.6	6 (1.08)	0.44–2.5
Protestant	382 (35.05)	32–38	75 (13.54)	11–17
Not religious	0 (0.00)	0.00–0.44	1 (0.18)	0.01–1.2
Race/Color				
White	134 (12.29)	10–14	83 (14.98)	12–18
Black	181 (16.61)	14–19	65 (11.73)	9.2–15
Indigenous/brown	775 (71.10)	68–74	406 (73.29)	69–77
Schooling				
High school completed	520 (47.71)	45–51	257 (46.39)	42–51
Elementary school not completed	5 (0.46)	0.17–1.1	4 (0.72)	0.23–2.0
Elementary school completed	24 (2.20)	1.4–3.3	11 (1.99)	1.0–3.6
High school not completed	64 (5.87)	4.6–7.5	29 (5.23)	3.6–7.5
College not completed	123 (11.28)	9.5–13	59 (10.65)	8.3–14
College completed	354 (32.48)	30–35	194 (35.02)	31–39
Income				
< 2 minimum wages	593 (58.89)	56–62	330 (63.83)	60–68
2–4 minimum wages	322 (31.98)	29–35	155 (29.98)	26–34
> 4 minimum wages	92 (9.14)	7.5–11	32 (6.19)	4.3–8.7

On average, the respondents engaged in 4.78 ± 1.41 types of activities (range: 0–6) and made 4.77 ± 1.16 types of home visits (range: 0–6). Most (77.91%) were frontline workers during the lockdown, despite not being trained for this type of work (83.96%). Slightly more than half (54.58%) reported having insufficient access to personal protective equipment (PPE), and 66.7% of the participants reported having insufficient biosafety norms in the workplace. Almost all (96.98%) believed they were susceptible to COVID-19 in the workplace (Table 2).

**Table 2:** Descriptive analysis of variables related to the work of community health workers (CHWs) according to location (state capitals vs. inland municipalities in Ceará).

Variables	State capitals		Inland municipalities	
	Mean (SD) or n(%)	95% CI	Mean (SD) or n(%)	95% CI
Types of activities (n)	4.65 (1.41)	4.6–4.7	5.05 (1.38)	4.9–5.2
Types of visits (n)	4.66 (1.14)	4.6–4.7	4.97 (1.17)	4.9–5.1
Covid-19 frontline work				
Yes	793 (73.09)	70–76	484 (87.36)	84–90
No	292 (26.91)	24–30	70 (12.64)	10–16
Received training to care for patients diagnosed with Covid-19				
Yes	126 (11.68)	9.9–14	136 (24.55)	21–28
No	953 (88.32)	86–90	418 (75.45)	72–79
Has access to PPE				
Yes	461 (42.53)	40–46	259 (46.75%)	43–51
No	623 (57.47)	54–60	295 (53.25)	49–57
Biosafety norms in the workplace are sufficient				
Yes	170 (15.61)	14–18	129 (23.29)	20–27
No	773 (70.98)	68–74	347 (62.64)	58–67
Doesn’t know	146 (13.41)	11–16	78 (14.08)	11–17
Susceptible to infection with coronavirus in the workplace				
Yes	1 054 (96.88)	96–98	538 (97.11)	95–98
No	34 (3.12)	2.2–4.4	16 (2.89)	1.7–4.7
Services were adapted to care for patients diagnosed with Covid-19				
Yes	816 (75.70)	73–78	407 (73.47)	70–77
No	262 (24.30)	22–27	147 (26.53)	23–30
Longer work hours to tend to patients diagnosed with Covid-19				
Yes	410 (37.89%)	35–41	382 (68.95%)	65–73
No	672 (62.11%)	59–65	172 (31.05%)	27–35
Capable of transmitting coronavirus in the workplace				
Yes	1 029 (94.58%)	93–96	522 (94.22%)	92–96
No	26 (2.39%)	1.6–3.5	13 (2.35%)	1.3–4.1
Not sure	33 (3.03%)	2.1–4.3	19 (3.43%)	2.1–5.4
Familiar with Covid-19				
Yes	814 (74.75%)	72–77	402 (72.56%)	69–76
No	237 (21.76%)	19–24	138 (24.91%)	21–29
Not sure	38 (3.49%)	2.5–4.8	14 (2.53%)	1.4–4.3
Was diagnosed with Covid-19				
Yes	437 (40.13%)	37–43	227 (40.97%)	37–45
Doesn’t know	126 (11.57%)	9.8–14	41 (7.40%)	5.4–10.0
No	526 (48.30%)	45–51	286 (51.62%)	47–56
Team work affected by the pandemic				
No	224 (20.57%)	18–23	126 (22.74%)	19–27
Yes	865 (79.43%)	77–82	428 (77.26%)	73–81

According to three quarters of the respondents (75.7%), the service was adapted to patients diagnosed with COVID-19, but 62.1% reported not having increased their workload. Moreover, almost all (94.46%) considered themselves potential vectors of coronavirus, 74% had one relative diagnosed with COVID-19, and 40.41% had themselves been diagnosed. The work processes changed during the pandemic according to 79.43% of the CHWs in the state capital and 77.26% of the CHWs in inland municipalities (Table 2).

Table 3 shows the variables related to the perception of violence, coronavirus anxiety, mental health, social support and QoL. Note that social relationships and support were positively associated with QoL in both state capitals (77%) and inland municipalities (74%).

**Table 3:** Descriptive analysis of continuous variables.

Variables	State capitals		Inland municipalities	
	Mean (SD) or n(%)	95% CI	Mean (SD) or n(%)	95% CI
Index of exposure—saw/heard about	0.56 (0.32)	0.54–0.58	0.38 (0.31)	0.35–0.40
Index of exposure—experienced	0.29 (0.28)	0.27–0.30	0.24 (0.27)	0.21–0.26
Coronavirus anxiety	0.80 (0.98)	0.74–0.86	0.66 (0.83)	0.60–0.73
SQR-20 score	6.68 (5.09)	6.4–7.0	6.27 (4.82)	5.9–6.7
SQR-20 groups				
Mental suffering	442 (41.19)	38–44	211 (38.09)	34–42
No mental suffering	631 (58.81)	56–62	343 (61.91)	58–66
MSPSS—family	5.51 (1.52)	5.4–5.6	5.75 (1.42)	5.6–5.9
MSPSS—friends	5.11 (1.53)	5.0–5.2	5.23 (1.49)	5.1–5.4
MSPSS—significant others	5.79 (1.44)	5.7–5.9	5.86 (1.35)	5.7–6.0
General self-efficacy (GSE) score	3.22 (0.54)	3.2–3.2	3.35 (0.51)	3.3–3.4
WHOQOL—physical health	3.48 (0.73)	3.4–3.5	3.62 (0.66)	3.6–3.7
WHOQOL—psychological health	3.75 (0.64)	3.7–3.8	3.83 (0.60)	3.8–3.9
WHOQOL—social relationships	3.71 (0.77)	3.7–3.8	3.78 (0.74)	3.7–3.8
WHOQOL—environment	3.17 (0.56)	3.1–3.2	3.37 (0.60)	3.3–3.4

F(25, 1297) = 68.22; *p* < 0.0001; R^2^ = 0.56; Adjusted R^2^ = 0.56.

The QoL of CHWs in state capitals was negatively influenced by low SRQ-20 scores, lack of training to care for patients diagnosed with COVID-19, perception of insufficient biosafety norms, and lack of adaptation of services to care for patients diagnosed with COVID-19. On the other hand, high MSPSS (family and friends) and high GSE were positively associated with QoL (Table 4).

**Table 4:** Predictors of QoL (WHOQOL) of community health workers (CHWs) working in state capitals (Fortaleza/CE, Recife/PE, Teresina/PI, João Pessoa/PB).

Predictors	Beta	95% CI	*p* value
Index of exposure—saw/heard about	0.02	− 0.06 to 0.10	0.7
Index of exposure—experienced	− 0.08	− 0.17 to 0.01	0.089
SQR-20 score	− 0.06	− 0.06 to − 0.05	**< 0.001**
MSPSS—family	0.04	0.01 to 0.06	**0.003**
MSPSS—friends	0.02	0.00 to 0.04	**0.016**
MSPSS—significant others	0.02	0.00 to 0.05	0.090
General self-efficacy (GSE) score	0.20	0.15 to 0.25	**< 0.001**
Time working for the ESF (Family Health Strategy)	0.00	0.00 to 0.00	0.8
Marital status			
Has no spouse/partner	–	–	
Has spouse/partner	− 0.04	− 0.09 to 0.01	0.15
Income			
< 2 minimum wages	–	–	
2–4 minimum wages	0.04	− 0.02 to 0.09	0.2
> 4 minimum wages	− 0.02	− 0.11 to 0.07	0.6
Received training to care for patients diagnosed with Covid-19			
Yes	–	–	
No	− 0.08	− 0.16 to 0.00	**0.049**
Biosafety norms in the workplace are sufficient			
Yes	–	–	
No	− 0.06	− 0.12 to 0.01	0.11
Doesn’t know	− 0.16	− 0.25 to − 0.07	** < 0.001**
Services were adapted to care for patients diagnosed with Covid-19			
Yes	–	–	
No	− 0.07	− 0.13 to − 0.01	**0.014**
Capable of transmitting coronavirus in the workplace			
Yes	–	–	
No	− 0.14	− 0.30 to 0.02	0.094
Not sure	0.09	− 0.05 to 0.22	0.2

F(19, 494) = 33.82; *p* < 0.0001; R^2^ = 0.56; Adjusted R^2^ = 0.54.

Significant values are in bold.

The QoL of CHWs in inland municipalities was negatively influenced by coronavirus anxiety, low SQR-20 scores, lack of training to care for patients diagnosed with COVID-19, lack of access to PPE, and lack of adaptation of services to care for patients diagnosed with COVID-19. On the other hand, high MSPSS (family and friends), high GSE, completed elementary school, and an income > 4 times the minimum wage were positively associated with QoL (Table 5).

**Table 5:** Predictors of QoL (WHOQOL) in inland municipalities (Sobral, Juazeiro do Norte, Crato, Barbalha).

Predictors	Beta	95% CI	*p*
Index of exposure—experienced	0.09	− 0.03 to 0.20	0.15
Coronavirus anxiety	− 0.06	− 0.11 to − 0.02	**0.004**
SQR-20 score	− 0.05	− 0.06 to − 0.04	**< 0.001**
MSPSS—family	0.07	0.04 to 0.09	**< 0.001**
MSPSS—friends	0.03	0.01 to 0.06	**0.014**
General self-efficacy (GSE) score	0.29	0.22 to 0.35	**< 0.001**
Age	0.00	− 0.01 to 0.00	0.4
Time working for the ESF (Family Health Strategy)	0.00	− 0.01 to 0.00	0.4
Schooling			
High school completed	–	–	
Elementary school not completed	0.25	− 0.10 to 0.60	0.2
Elementary school completed	0.35	0.11 to 0.59	**0.004**
High school not completed	− 0.05	− 0.20 to 0.11	0.5
College not completed	− 0.07	− 0.18 to 0.04	0.2
College completed	− 0.02	− 0.09 to 0.05	0.5
Income			
< 2 minimum wages	–	–	
2–4 minimum wages	0.00	− 0.07 to 0.07	> 0.9
> 4 minimum wages	0.18	0.05 to 0.31	**0.006**
Received training to care for patients diagnosed with Covid-19			
Yes	–	–	
No	− 0.08	− 0.15 to 0.00	**0.039**
Has access to PPE			
Yes	–	–	
No	− 0.08	− 0.14 to − 0.01	**0.018**
Services were adapted to care for patients diagnosed with Covid-19			
Yes	–	–	
No	0.08	0.00 to 0.15	**0.049**
Longer work hours to tend to patients diagnosed with Covid-19			
Yes	–	–	
No	− 0.07	− 0.14 to 0.00	0.065

F(23, 490) = 28.56; *p* = 0.0001; R^2^ = 0.57; Adjusted R^2^ = 0.55.

Significant values are in bold.

## Discussion

In this study, we evaluated how the QoL of Brazilian CHWs was impacted by adverse working conditions during the pandemic in different contexts and territories (four northeastern state capitals and four inland municipalities). Our findings can foster debates on how to better prepare health care services and make systems more resilient to future social and sanitary challenges. In health care, resilience is defined as the ability of health workers, administrators, institutions and communities to maintain essential services while running during times of adversity and reorganize structures and services based on lessons learned during crises^40^.

The predominance of the female sex in the field work force matches the findings of other studies^41,42^. However, it should be noted that many workers also tend to have their homes and families, and the burden and stress associated with multiple functions and insufficient rest can take their toll on health^43^. It is important to emphasize the multiple roles culturally assumed by the female universe, as mother, daughter, partner, which, in the face of the risk of transmissibility, have aggravated the burden due to the necessary behavioral changes both in the work environment and in the family. This may be exacerbated by the prevailing culture, according to which women are assumed to be more aware/sensitive to the needs of others^44^ and thus under more pressure to be the source of care, which in turn may affect their quality of life. In terms of gender inequalities affecting quality of life, UN Women Brazil concluded that the pandemic has worsened living and working conditions and increased cases of gender-based violence against women and girls^45^.

In this study, social support (family and friends) and general self-efficacy were associated with better QoL in both scenarios (state capitals and inland municipalities). In another study, family support was found to favor self-efficacy with regard to the control and prevention of diabetes and asthma episodes^46,47^. Whether in crisis or not, human behavior is modulated by the meaning individuals assign to their interactions with others, themselves and the social environment^15^.

Family and friends had a greater positive impact than ‘significant others’, which is consistent with the findings of other researchers^20,21,31,48^ and indicates the potential of these networks as a source of social support for CHWs. Thus, strategies favoring and expanding social support should be implemented, among other things, by creating space for the exchange of experiences, improving working conditions, implementing occupational health actions through psychosocial support programs, and strengthening collaboration and teamwork skills^49^. Such initiatives are likely to mitigate the negative effects of sanitary emergencies on CHWs’ mental health and QoL.

Social support reinforces CHWs’ perceptions of belonging, acceptance, appreciation, care and love—a feeling crucial in times of increased stress, crisis and peril^20,37^. The restrictions imposed during the recent lockdown heavily affected family and social relationships and led to significant changes in health work routines. In such situations, social support networks contribute to preserving QoL.

The data collected from the state capitals show that a lack of adaptation of services to care for patients diagnosed with COVID-19 and lack of adequate training led to a loss of QoL in this setting. In the inland municipalities, lack of training, lack of adaptation of services, and unavailability of PPE also negatively impacted QoL.

The measures carried out to combat Covid-19 varied among the eight sampling locations due to differences in sociodemographic, economic and cultural profiles; the capacity of each local health care infrastructure; and the availability of resources and personnel. However, a set of commonalities was observed, especially with regard to the resilience of the local health care system. Professional insecurity resulting from living with urban violence implies deficits in the development of actions outside the health unit (such as home visits and educational activities) and possible suspension/termination of consultations within teams^50–52^, aggravating the relationship with the community and could have also affected professionals’ QofL.

Our results showed that the working conditions of the CHWs in both state capitals and inland municipalities were unsatisfactory during the health emergency. In the same period, the public health care sector was marred by administrative difficulties and protracted political conflicts, with potentially serious repercussions on morbidity and mortality rates^7,23,53^. The health work process has been greatly compromised, the responses to the challenges imposed by covid-19 have been heterogeneous, which has had repercussions on the quality of life of health professionals^54,55^. A study showed that in Brazil, doctors (69.5 per cent) and nursing staff (64.1 per cent) and community health workers and endemic control workers (34.1 per cent) received personal protective equipment (PPE) continuously during the pandemic^56^.

In Brazil, local health authorities, rather than the federal government, managed the response to the pandemic. Immediate safety measures and new processes had to be devised to address unusual disease patterns and deaths. In several studies, health professionals have reported receiving insufficient training and information^7,23,56,57^. In the absence of a unified national response, problems at the municipal level often delay the implementation of effective health actions.

Uncertainty about the new work procedures required during sanitary emergencies had a negative effect on the QoL of the CHWs. This experience is important to keep in mind when designing new local interventions and public policies that incorporate the demands and needs of CHWs. Moreover, the QoL of other Brazilian health care workers, such as nurses, was equally affected during this period^20,58,59^.

The capacity of the public secondary and tertiary-level health care infrastructure was highly taxed, sometimes overburdened, during this period. Control measures at the primary care level and the occupational safety of the CHWs were not part of the initial action plan. This is reflected in a review^60^ of public health care measures covering the period 2019–2020: the twenty most relevant topics included health workers’ mental health (5th place), health worker and patient safety (9th place), and training/distance learning (10th place). Primary care was not included among the twenty topics.

The workload of CHWs in territories, employing their know-how and experience to bridge the gap between the community and the health care system, escalated during lockdown, affecting their QoL and increasing their risk of developing CMDs. This harmful effect was to some degree felt in society as a whole as the inevitable result of the sanitary emergency, fear of exposure to a novel illness, and the flow of reports of increased mortality^20^.

Health workers are exposed to various types of violence, including living with, interacting with, and witnessing situations of violence in the area, through direct or indirect contact with victims of violence or aggressors^61^. Faced with this scenario, CHWs are the most exposed professionals, since they directly perform functions outside the health unit, such as home visits and active searches, and are vulnerable to threats during the community approach^61–64^. CHWs represent the health facility and, hence, the State. Nevertheless, they are also under pressure from a local parallel power, with unknown rules. Thus, the daily activities of CHWs occur in a territory under a precarious balance between the power of the State and the power of local gangs^65^ where CHWs are most inhibited in territories where the State has less control and where health challenges are particularly severe^9^. The consequences of victimization in the workplace include psychosomatic symptoms and can manifest themselves in the form of feelings of distrust, discouragement, loss of meaning in work, stress, anxiety, isolation, low self-esteem, among others^66^, which tend to worsen over time, which can lead to disruption of interpersonal relationships, absenteeism, turnover, illness, absenteeism, disruption of work organization^67^ and possibly professional quality of life. Thus, the importance to evaluate quality of life of CHW on the context of stressors, such as urban violence and Covid-19.

The lessons learned during this period will help strengthen the public health care system and improve the management and support of frontline workers. Job burnout has serious implications for health, employee retention and the work environment. Policies are urgently needed to protect the health and QoL of health care workers and, at the same time, to increase the resilience of the health care system.

Methodologically speaking, the present study was limited by its exclusive reliance on self-reported data, which introduced some degree of subjectivity and memory bias in the participants’ responses. Additionally, the cross-sectional study design adopted precluded an analysis of causality.

The effects of the COVID-19 pandemic on the QoL of CHWs require long-term monitoring. Studies on sanitary emergencies have generally paid little attention to primary care workers’ health and occupational safety, but the effects are gradually becoming more perceptible.

## Conclusions

The long-term effects of the stressful context of violence and health emergencies on the QoL of CHWs are not yet fully understood and require longitudinal evaluations. Nevertheless, the decrease in QoL of Brazilian frontline CHWs associated with health emergencies identified in the present study has highlighted the need to improve working conditions and procedures. Respondents reported high levels of CMDs, which were mitigated by social support outside the workplace. The health, well-being, and quality of life of CHWs are essential to their role as a link between the community and the health system, and to making the health system more resilient and efficient.

The health crisis has sparked a social emergency, prompting significant shifts in the professional landscape that have impacted workers across all levels of care, affecting their quality of life. This impact has been particularly evident in Primary Health Care (PHC), which stands as the frontline of community care. The post-traumatic effects experienced by workers, stemming from societal pressures, underscore the critical need to address two key aspects of workers' well-being within the PHC setting.

Firstly, there's a clear imperative to establish and maintain a psychosocial support service tailored to the needs of PHC workers within their workplace. Secondly, it's essential to bolster social interactions within interprofessional teams through dialogic and participatory communication among team members and the community. This approach is vital for fostering a supportive environment and ultimately enhancing the quality of life for the PHC workforce.

## Data Availability

The datasets used and/or analyzed during the current study are available from the corresponding author upon reasonable request.
